# Depletion of SAG/RBX2 E3 ubiquitin ligase suppresses prostate tumorigenesis via inactivation of the PI3K/AKT/mTOR axis

**DOI:** 10.1186/s12943-016-0567-6

**Published:** 2016-12-12

**Authors:** Mingjia Tan, Jie Xu, Javed Siddiqui, Felix Feng, Yi Sun

**Affiliations:** 1 Division of Radiation and Cancer Biology, Department of Radiation Oncology, 4424B MS-1, 1301 Catherine Street, Ann Arbor, 48109 MI USA; 2 Institute of Translational Medicine, Zhejiang University School of Medicine, Hangzhou, Zhejiang People’s Republic of China; 3 Collaborative Innovation Center for Diagnosis and Treatment of Infectious Diseases, Zhejiang University, Hangzhou, People’s Republic of China; 4 Department of Pathology, University of Michigan, 4424B MS-1, 1301 Catherine Street, Ann Arbor, 48109 MI USA; 5 Department of Radiation Oncology, University of San Francisco, San Francisco, CA USA

**Keywords:** Prostate tumorigenesis, Pten, PHLPP1, DEPTOR, SAG KO, SAG-SCF E3, Ubiquitin ligase

## Abstract

**Background:**

SAG (Sensitive to Apoptosis Gene), also known as RBX2, ROC2 or RNF7, is a RING component of CRL (Cullin-RING ligase), required for its activity. Our recent study showed that SAG/RBX2 co-operated with Kras to promote lung tumorigenesis, but antagonized Kras to inhibit skin tumorigenesis, suggesting a tissue/context dependent function of Sag. However, it is totally unknown whether and how Sag would play in prostate tumorigenesis, triggered by Pten loss.

**Methods:**

*Sag* and *Pten* double conditional knockout mice were generated and prostate specific deletion of *Sag* and *Pten* was achieved by PB4-Cre, and their effect on prostate tumorigenesis was evaluated by H&E staining. The methods of immunohistochemistry (IHC) staining and Western blotting were utilized to examine expression of various proteins in prostate cancer tissues or cell lines. The effect of SAG knockdown in proliferation, survival and migration was evaluated in two prostate cancer cell lines. The poly-ubiquitylation of PHLPP1 and DEPTOR was evaluated by both in vivo and in vitro ubiquitylation assays.

**Results:**

SAG is overexpressed progressively from early-to-late stage of human prostate cancer with the highest expression seen in metastatic lesion. *Sag* deletion inhibits prostate tumorigenesis triggered by *Pten* loss in a mouse model as a result of suppressed proliferation. SAG knockdown in human prostate cancer cells inhibits a) proliferation in monolayer and soft agar, b) clonogenic survival, and c) migration. SAG is an E3 ligase that promotes ubiquitylation and degradation of PHLPP1 and DEPTOR, leading to activation of the PI3K/AKT/mTOR axis, whereas SAG knockdown caused their accumulation. Importantly, growth suppression triggered by SAG knockdown was partially rescued by simultaneous knockdown of PHLPP1 or DEPTOR, suggesting their causal role. Accumulation of Phlpp1 and Deptor with corresponding inactivation of Akt/mTOR was also detected in Sag-null prostate cancer tissues.

**Conclusions:**

*Sag* is an oncogenic cooperator of *Pten*-loss for prostate tumorigenesis. Targeting SAG E3 ligase may, therefore, have therapeutic value for the treatment of prostate cancer associated with *Pten* loss.

**Electronic supplementary material:**

The online version of this article (doi:10.1186/s12943-016-0567-6) contains supplementary material, which is available to authorized users.

## Background

Prostate cancer is one of the most common malignancies and the second leading cause of cancer death in males [1]. It develops through successive stages including intra-epithelial neoplasia (PIN), carcinoma in situ, invasive adenocarcinoma, and metastatic diseases [2]. The disease is complex in its development and response to therapy, and it cannot be predicted when or whether an indolent prostate tumor will be become clinically aggressive. Furthermore, the limitations in current treatment methods warrant an intense focus on this type of cancer. Finally, the development of effective targeted therapies will require a better understanding of the signaling cascades responsible for the initiation and progression of prostate cancer.

SCF (SKP1, Cullin1 and F-box protein) E3 ligase, also known as CRL1 (Cullin RING ligase), the founding member of CRLs, promotes the ubiquitylation and degradation of various key regulatory proteins, thus controlling several important biological processes including cell cycle progression, signal transduction, transcription, DNA replication, tumorigenesis and angiogenesis [3–7]. The SCF consists of four components: an adaptor protein Skp1, a scaffold protein cullin, an F-box protein, and a RING protein [3, 4]. Whereas the human genome encodes 69 F-box proteins [8, 9] that confers substrate specificity, there are only two RING family members of RING proteins in human or mouse, RBX1/ROC1, and SAG/RBX2/ROC2/RNF7 [6, 10–12]. It is established that RBX1/ROC1 prefers to bind to cullin family members, CUL 1–3 and Cul4A/B, and SAG/RBX2 prefers to bind to CUL5, as well as CUL1 [13–17]. While biochemically, RBX1 and SAG are interchangeable for E3 ligase activity [18, 19]; our KO study revealed that biologically, they are NOT functionally redundant during mouse embryonic development. *Rbx1* KO in a wt *Sag* background causes embryonic death at E7.5 with p27 accumulation [20]; where *Sag* KO in a wt *Rbx1* background also causes embryonic death, but at E10.5–11.5 with NF1 accumulation [17], suggesting that the two proteins have unique sets of substrates for degradation in vivo. Sag endothelial deletion also causes embryonic lethality at a later stage around E15.5 with defective vasculogenesis and endothelial cells proliferation [7]. In human tissues, SAG overexpression was detected in carcinomas of lung, colon, stomach, cervix and liver, with poor survival of lung cancer patients [21–25]. Furthermore, *Sag* transgenic expression regulated skin tumorigenesis induced by DMBA-TPA [26], and UVB-radiation [27], whereas *Sag* deletion in mouse embryonic fibroblasts suppressed *Kras* ^*G12D*^ *–*induced immortalization and transformation [28]. More interestingly, Sag played a tissue- and context-dependent oncogenic or tumor suppressive role in *Kras* ^*G12D*^-driven mouse tumorigenesis. While *Sag* deletion in the lung significantly reduced lung tumorigenesis [25], it accelerated skin tumorigenesis when deleted in the skin [29]. However, it is unknown whether Sag plays a role in prostate tumorigenesis, and, if so, what is the underlying mechanism.

The *Pten*, a non-redundant gene encoding a phosphatase, is frequently deleted or mutated in human cancer [30]. Loss of PTEN in human cancer cell lines and mouse models results in constitutive activation of the PI3K/AKT pathway, leading to enhanced cell growth and survival [31]. *Pten* homozygous deletion in mice causes early embryonic death, and *Pten* heterozygous mice exhibit hyperplastic-dysplastic changes in multiple organs, including PIN in mouse prostate without progression to adenocarcinoma [32]. Conditional homozygous deletion of *Pten* in mouse prostate significantly shortens the latency of PINs and promotes their progression to metastatic cancer characteristic of human prostate cancer [33].

Several phosphatases negatively regulate the PI3K/AKT pathway. Two isoforms of PHLPP, PHLPP1 and PHLPP2, have been shown to directly dephosphorylate AKT [34]. PHLPP1 and PHLPP2 are reported to be lost in 30% and 50% of prostate cancer, respectively, highlighting their clinical importance [34]. PHLPP1 protein is ubiquitylated by SCF^β-TrCP^ E3 ubiquitin ligase for subsequent degradation by proteasome [35].

DEPTOR was identified as a naturally occurring inhibitor of both mTORC1 and mTORC2 [36]. In cell culture settings, DEPTOR mainly acts as a tumor suppressor, since its loss activates mTORC1 and mTORC2 to promote growth and survival of cancer cells [36]. Recently, we, along with other two groups, found that DEPTOR is yet another substrate of SCF^β-TrCP^ E3 ligase [37–39].

In this study, we used the *Sag* conditional KO mouse model in combination with the *Pten* loss in prostate to determine the in vivo role of *Sag* in prostate tumorigenesis. We found that the *Sag* deletion suppressed the progression of prostate cancer induced by *Pten*-loss with mechanism involving accumulation of Phlpp1 and Deptor to inhibit PI3K/AKT/mTOR signaling pathway. Consistently, SAG knockdown suppressed the growth and survival of human prostate cancer cells due to accumulation of PHLPP1 and DEPTOR, two new substrates of SAG E3 ligase, which can be partially rescued by simultaneous knockdown of PHLPP1 or DEPTOR. Thus, Sag appears to act as an oncogenic gene cooperating with *Pten*-loss to promote prostate tumorigenesis by activating the PI3K/AKT/mTOR signaling pathway.

## Methods

### Reagents

We purchased antibodies against p21 (mouse mAb) and p27 (mouse mAb) from BD Transduction Labs (Gibbstown, NJ), antibodies against DEPTOR, AKT, pS6, 4EBP1, p4EBP1 cleaved Casp3 and pAKT polyclonal antibodies from Cell Signaling Technology (Danvers, MA), S6 (mouse mAb) and NRF2 from Santa Cruz Biotechnology (Santa Cruz, CA), PHLPP1 polyclonal antibody from Bethyl Laboratories (Montgomery, TX) and β-Actin (mouse mAb) from Sigma (St. Louis, MO), Ki67 from BD Biosciences (Gibbstown, NJ), Dab1 and BrdU (rat mAb) from Abcam (Cambridge, MA), FLNA from Abnova (Walnut, CA). DEPTOR siRNA, PHLPP1 siRNA and control siRNA were obtained from Santa Cruz Biotechnology (Santa Cruz, CA). In Situ Cell Death Detection Kit was purchased from Roche (Indianapolis, IN). ATPlite kit was obtained from Perkin Elmer (Boston, MA).

### Cell cultures

Human prostatic carcinoma cell lines, Du145 and PC3 were purchased from the American Type Culture Collection (ATCC, Manassas, VA) and cultured in standard RPMI 1640 medium containing 10% fetal bovine serum (Invitrogen, Carlsbad, CA), at 37 °C under a humidified atmosphere of 95% air and 5% CO_2_.

### Mouse studies

The *Sag* ^*fl/fl*^ conditional KO mouse model was generated with exon 1 flanked with loxP sites [28]. Pb4-Cre and *Pten* ^*fl/fl*^ (strain B6.129S4-Pten^tm1Hwu^/J) mice were purchased from Jackson laboratories. All procedures were approved by the University of Michigan Committee on Use and Care of Animals. Animal care was provided in accordance with the principles and procedures outlined in the National Research Council Guide for the Care and Use of Laboratory Animals.

### PCR-based genotyping

Genomic DNA was isolated from mouse tail tips and was genotyped using the primer set of PSag-KO-F: 5’-TTCTGGCCAGGTGTGGTGATATC-3’, and PSag-KO-G: 5’-CTTAGCCTT GGTTGTGTAGAC-3’ to detect floxed allele (140 bp) and wild type allele (105 bp) of *Sag*. The primer set for *Pten* is oIMR9554: 5’-CAAGCACTCTGCGAACTGAG-3’ and oIMR9555: 5’-AAGTTTTTGAAGGCAAGATGC-3’ to detect floxed allele (328 bp) and wild type allele (156 bp). The primer set for PB4-Cre is PB4-Cre-C001: 5’-ACCAGCCAGCTATCAACTCG-3’, PB4-Cre-C002: 5’-TTACATTGGTCCAGCCACC-3’, PB4-Cre-C003: 5’-CTAGGCCACAGA ATTGAAAGATCT-3’ and PB4-Cre-C004: 5’-GTAGGTGGAAATTCTAGCATCATCC-3’ to detect Cre (260 bp) and wild type allele (400 bp), respectively.

### Immunoblotting analysis

Human prostate cancer cells or mouse prostate tissues were harvested, lysed in a Triton X-100 lysis buffer (20 mM Tris/HCl pH 8.0, 150 mM NaCl, 1% Triton X-100, 5 mM EDTA and 5 mM EGTA) supplemented with protease inhibitor cocktail (Roche). Lysates were incubated on ice for 30 min, centrifuged (13,000 r.p.m, 15 min, 4 °C) and supernatants were subjected to SDS-PAGE. Gels were transferred to nitrocellulose membranes. Membranes were blocked with 5% milk in TBST (50 mM Tris/HCl, pH 7.4, 150 mM NaCl, 0.1% Tween 20) and incubated with primary antibodies of interest in 5% milk in TBST overnight at 4 °C, and then with horseradish peroxidase-conjugated secondary antibodies for 1 h at RT. Samples were visualized with enhanced chemiluminescence and X-ray film. SAG monoclonal antibody was raised against the RING domain (AA44-113) [23].

### Histology and Immunohistochemistry staining

Prostate tissues were fixed in 10% formalin and embedded in paraffin. Five-μm-thick sections were cut for H&E staining and examined under a microscope. Prostate hyperplasia is characterized by proliferation of luminal cells without cytological atypia, but containing small foci with two or three layers of cells. The PIN lesions were graded using the nomenclature and criteria developed by Park JH, et al [40]. In brief, high-grade PIN is characterized by an intraglandular proliferation of crowding cells with atypia, and cribriform formation or the development of multi-layered solid glandular structures. Invasive adenocarcinoma is characterized by proliferation of atypical cells that break the basal membrane and invade through the prostatic stroma. Immunohistochemistry was performed using the ABC Vectastain kit (Vector Laboratories, Burlingame, CA) with antibodies against SAG monoclonal antibody, Ki67 (1:1000), BrdU (1:1000), AR (1:500), DEPTOR (1:500), cleaved caspase3 (1:200), pAkt (1:500), Pten (1:500), p4EBP1(1:1000) and pS6 (1:500) and PHLPP1 (1:500). The sections were developed with DAB and counterstained with haematoxylin.

### siRNA knockdown

The lenti-virus-based siRNA knockdown of SAG (Lt-SAG, 5’-GAGGACUGUGUUGU GGUCU-3’), along with scrambled siRNA control (Lt-Con, 5’-AUUGUAUGCGAUC GCAGAC-3’) was performed as described [7]. For double silencing, cells were infected with Lt-Sag or Lt-Con for 48 to 72 hrs in 60-mm dishes. Cells were then split into 60-mm dishes and transiently transfected with si-Con or Si-PHLPP1 (siRNA pools from Santa Cruz Biotechnology, Santa Cruz, CA) or Si-DEPTOR (5’-GCCATGACAATCGGAAATCTA-3’) using Lipofectamine 2000 (Life Technology, Carlsbad, CA). Forty-eight hours later, cells were harvested for proliferation, clonogenic, migration and soft agar assay.

### Migration assay

Human prostate tumor cells infected with Lt-Con or Lt-SAG were subjected to Boyden chamber migration assay (BD Biosciences, Gibbstown, NJ) according to manufacturer’s instruction.

### ATPlite-based cell proliferation assay

Cells were seeded in 96-well plates in triplicates and cell proliferation was measured with an ATPlite kit (Perkin Elmer, Boston, MA) [41].

### Clonogenic and soft agar assays

Cells after lenti-virus-based shRNA silencing or siRNA oligonucleotide transfection were seeded in 60 mm dishes in triplicate followed by incubation at 37 °C for 9 to 14 days. The colonies formed were stained and counted under microscope. Soft agar assay was performed as described [42].

### TUNEL assay

Prostate tissues were fixed in 10% formalin and embedded in paraffin. Five-μm-thick sections were cut for assessing for apoptosis by TUNEL assay using the In Situ Cell Death Detection Kit (Roche, Indianapolis, IN), according to the manufacturer’s recommendations.

### The In vitro ubiquitination assay

Cullin1-SAG E3 complex was precipitated from 293 cells overexpressing both proteins with FLAG tags. HA-tagged PHLPP1 or HA-tagged PHLPP1(4A) was pulled down by HA-conjugated beads after transfection into 293 cells and eluted with 1× HA peptide (Roche, Indianapolis, IN). FLAG-tagged DEPTOR or FLAG-Tagged DEPTOR(3A) also was pulled down by FLAG beads and eluted with 3× FLAG peptide (Sigma, St. Louis, MO), followed by incubation with Cullin-SAG E3 complex in the presence of E1 and E2 in a ubiquitin reaction buffer. Polyubiquitylated PHLPP1 or DEPTOR was resolved by SDS-PAGE and detected using antibody against PHLPP1 or DEPTOR, respectively.

### The in vivo ubiquitylation assay

The 293 cells were cotransfected with HA-PHLPP1, Cullin, SAG and His-Ub or FLAG-DEPTOR, Cullins, SAG and His-Ub. Thirty-six hours post transfection, cells were lysed in 6 M guanidinium denaturing solution, as described previously [43]. Poly-ubiquitylated proteins was purified by Ni-bead pull-down and detected by IB using PHLPP1 or DEPTOR antibody, respectively.

### Statistical analysis

Statistical analysis was performed using two-tailed student’s *t*-test. All statistical analyses were carried out using the GraphPad Prism software version 5.01 (GraphPad, San Diego, CA). Data were expressed as mean ± standard error of the mean (SEM) of at least 3 independent experiments. A *P* value < 0.05 was considered statistically significant.

## Results

### SAG is overexpressed in human prostate cancer tissues, whereas Sag prostate knockout inhibits tumorigenesis induced by *Pten* loss due to reduced proliferation

**Fig. 1:**
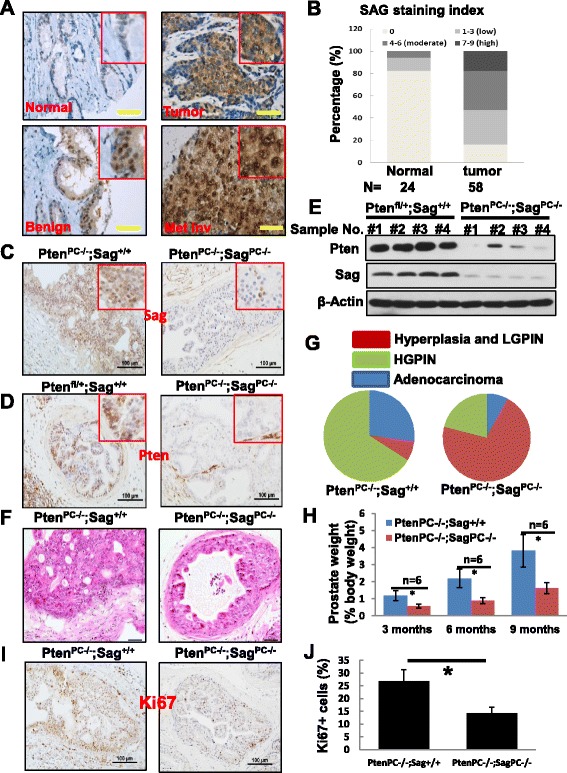
SAG is crucial for progression of prostate cancer in human and mice. (**a** & **b**) Expression of SAG is assessed in a human prostate cancer TMA. SAG staining indexes in cohorts of normal prostate epithelial cells (*n* = 24) and prostate tumors (*n* = 58) are shown as stacked columns. (**c** & **d**) PB4-Cre inactivates Pten and Sag in prostate epithelium cells. Prostate tissues from mice at age of 6 months with indicated genotypes were fixed in 10% formalin, embedded, sectioned and stained with Pten and Sag Abs. (**e**) Western blotting for Pten and Sag in prostate tissues (four independent samples with two genotypes). (**f**) Haematoxylin and eosin (**h** & **e**) staining of the prostate tissues from mice at age of 6 months with indicated genotypes. Scale bar represents 100 μm. (**g**) Pie graphs show prostate tumor progressions in *Pten* ^*PC*-/-^;*Sag* ^+/+^ and *Pten* ^*PC*-/-^;*Sag* ^*PC*-/-^ mice at age of 6 months (*n* = 10). HGPIN, high-grade PIN; LGPIN, low-grade PIN. The quantitative results of tumor progression are from two randomly selected slides of each mouse in a total 10 pairs of the entire animal cohort. (**h**) Mice with indicated genotypes were sacrificed at ages of 3, 6, and 9 months, respectively. The weight of prostate vs. whole body were weighed, the % weight was calculated and plotted (*n* = 6). * *p* < 0.05. (**i** & **j**) Prostate tissues with indicated genotypes were stained for Ki67 with representative images shown (**i**). Positive cells were counted from at least 3 randomly selected microscopic fields with % positivity calculated (**j**). * *p* < 0.05. Scale bar: 100 μm

Our recent study showed that both RBX1 and SAG/RBX2 are overexpressed in human non-small cell lung carcinomas [23, 44]. While overexpression of SAG, but not of RBX1, is associated with poor survival of lung cancer patients, SAG expression is required to lung tumorigenesis triggered by Kras mutation [25]. To determine potential role of Sag in other human cancers, we measured SAG levels in prostate tissue microarray consisting of normal (n = 24) versus tumor samples (n = 58) and found that SAG expression was progressively increased from normal to benign, then to malignant lesions with the highest expression seen in metastatic tumors (Fig. 1a & b).

To determine whether SAG overexpression is causally related to, or merely the consequence of prostate tumorigenesis, we crossed *Sag* ^*fl/fl*^ mice with *Pb4-Cre*;*Pten* ^*fl/fl*^ mice, a well-established prostate cancer model in which *Pten* ^*fl*/*fl*^ is deleted specifically in prostate epithelial cells by Pb4 driven Cre-recombinase to induce epithelial hyperplasia, adenomas and adenocarcinomas [33]. Two rounds of crossing gave rise to the compound mice with the following two genotypes: 1) *Pb4-Cre*;*Pten* ^*fl*/*fl*^;*Sag* ^+/+^ *(Pten* ^*PC-*/*-*^;*Sag* ^+/+^) and 2) *Pb4-Cre*;*Pten* ^*fl*/*fl*^;*Sag* ^*fl*/*fl*^ *(Pten* ^*PC-*/*-*^;*Sag* ^*PC*-/-^ *)*. We first measured the expression of Sag and Pten in prostate tissues from paired mice at age of 6 months by immuno-histochemical staining (Fig. 1c & d) and western blotting (Fig. 1e), and confirmed a significant reduction of both Pten and Sag levels in *Pten* ^*PC*-/-^;*Sag* ^*PC*-/-^ prostates. We then euthanized paired mice at age of 3, 6, and 9 months, followed by examination for prostate lesions. At age of 6 month, both *Pten* ^*PC*-/-^;*Sag* ^+/+^ and *Pten* ^*PC*-/-^;*Sag* ^*PC*-/-^ mice developed various lesions, ranging from hyperplasia, low grade intraepithelial neoplasia (LGPIN), high grade intraepithelial neoplasia (HGPIN), as well as adenocarcinoma. Quantitative analysis of prostate tissues, however, revealed that *Sag* deletion significantly reduced prostate tumor burden, as evidenced by significant reduction of incidence of HGPIN and adenocarcinoma, with a majority of cases being hyperplasia and LGPIN (Fig. 1f&g). Consistently, the weight ratio of prostate vs. whole body was significantly lower in *Pten* ^*PC*-/-^;*Sag* ^*PC*-/-^ than in *Pten* ^*PC*-/-^;*Sag* ^+/+^ mice for all three groups of mice at ages of 3 (0.58% vs. 1.18%), 6 (0.89% vs. 2.19%) and 9 (1.62% vs. 3.82%) months (Fig. 1h). It is worth noting that *Sag* deletion alone has no effect on normal development of prostate tissues, since no prostate lesions were observed at all groups of mice at ages of 3, 6, and 9 months (data not shown). Collectively, these results strongly suggest that *Sag* inactivation may not affect tumor initiation, but could remarkably inhibit the disease progression from hyperplasia to adenocarcinomas. Thus, *Sag* appears required for the progression of prostate tumorigenesis, triggered by *Pten* loss.

To define the nature by which *Sag* deficiency suppressed the development of prostate cancer, we determined the effect of Sag on proliferation and apoptosis by immuno-staining the prostate tissues from 6 month-old mice with genotypes of *Pten* ^*PC*-/-^;*Sag* ^*PC*-/-^ vs. *Pten* ^*PC*-/-^;*Sag* ^+/+^ with proliferation markers Ki67 and BrdU, and apoptosis markers caspase-3 and TUNEL. Remarkably, Sag deletion significantly reduced the overall proliferation, as stained by Ki67 (Fig. 1i&j) as well as the rate of proliferation, as evidenced by reduced BrdU labelling (Additional file 1: Figure S1A&B), but had no effect on apoptosis (Additional file 1: Figure S1C-F). Reduced proliferation was also observed in the prostate tissues in *Pten* ^*PC*-/-^;*Sag* ^*PC*-/-^ mice at age of 3 or 9 months (data not shown). Thus, *Sag* inactivation likely suppressed prostate tumorigenesis by inhibiting cell proliferation.

### SAG knockdown inhibits the growth, survival and migration of human prostate cancer cells via inducing accumulation of PHLPP1 and DEPTOR to inactivate PI3K/AKT/mTOR pathway

**Fig. 2:**
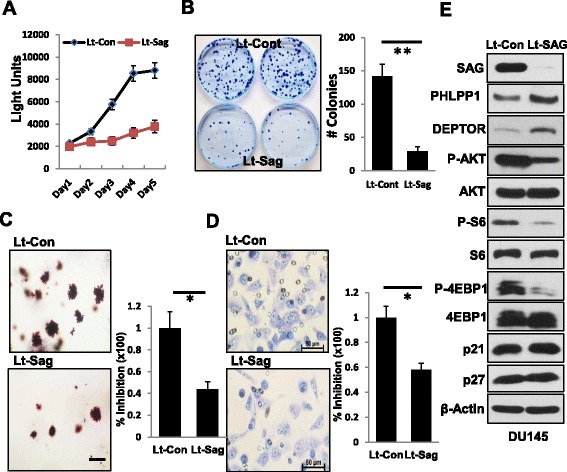
SAG Knockdown suppresses growth, survival and migration of human prostate cancer cells via inactivation of the PI3K/AKT/mTOR axis. DU145 human prostate cancer cells were infected with Lenti-SAG (Lt-SAG), along with Lenti-GFP (Lt-Con) as a control for 72 hrs. Cell proliferation was measured by ATP-lite assay (**a**), clonogenic survival (**b**), soft agar assay (**c**), and Boyden chamber migration assay (**d**), as well as western blotting assay using indicated Abs (**e**). Shown are mean ± SEM from three independent experiments (**a**-**d**). * *p* ; ** *p* < 0.01. Scale bar represents 50 μm

Having established that *Sag* is required for prostate tumorigenesis in Pten loss mouse model *in vivo*, we next used *in vitro* cell culture models to further investigate the role of SAG in the growth and survival of human prostate cancer cells. We used two human prostate cancer cell lines DU145 and PC3. Indeed, SAG knockdown via lentivirus-based siRNA silencing caused significant reduction in A) monolayer growth; B) clonogenic survival; and C) anchorage-independent growth in soft-agar (Fig. 2a-c, and Additional file 1: Figure S2A-C). Furthermore, SAG knockdown also significantly inhibited migration of human prostate cancer cells (Fig. 2D and Additional file 1: Figure S2D). Therefore, Sag is required for the growth and survival of prostate cancer cells as well as for the maintenance of the tumor cell phenotype.

To explore the potential mechanisms by which SAG knockdown suppresses the growth of prostate cancer cells. We determined potential accumulation of naturally occurring inhibitors of the PI3K/AKT/mTOR axis, known to be the substrates of SCF E3 ligase [6]. SAG knockdown caused accumulation of PHLPP1 (Fig. 2e and Additional file 1: Figure S2E), a protein phosphatase which directly dephosphorylates and inactivates AKT [45], also a known physiological substrate of SCF^β-TrCP^ [35]. We also found that SAG knockdown caused accumulation of DEPTOR, an mTOR inhibitor [36] and a physiological substrate of SCF^βTrCP^ [37–39] as well, with consequent inactivation of mTOR signals, as reflected by reduced phosphorylation of S6 and 4EBP1 (Fig. 2e and Additional file 1: Figure S2E). Interestingly, the accumulation of CRL substrates induced by SAG knockdown was rather selective, since other two SCF/CRL1 substrates p21 and p27 (Fig. 2e and Additional file 1: Figure S2E), two CRL5 substrates FLNA and DAB1, and one CRL3 substrate NRF2 (Additional file 1: Figure S3) were not accumulated in these two lines of prostate cancer cells upon SAG depletion. Collectively, our results suggested that SAG knockdown triggers accumulation of PHLPP1 and DEPTOR to inactivate the AKT/mTOR axis, leading to observed suppression of growth and survival in prostate cancer cells.

### SAG forms a complex with PHLPP1 or DEPTOR, shortens their protein half-life by promoting their ubiquitylation

**Fig. 3:**
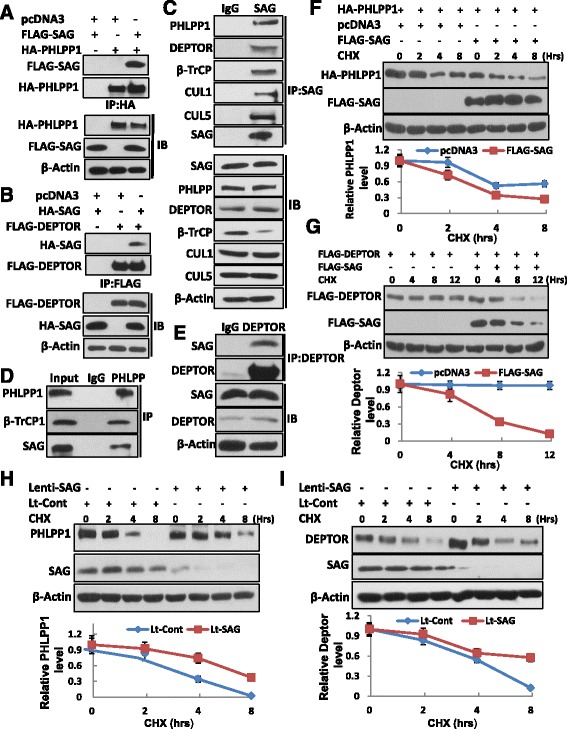
SAG interacts with PHLPP1 or DEPTOR and shortens their protein half-lives. (**a**-**e**) SAG interacts with PHLPP1 or DEPTOR: DU145 cells were co-transfected with indicated plasmids, cell lysates were prepared and IP with anti-HA Ab, IB with anti-FLAG Ab (**a**), or IP with anti-FLAG Ab, IB with anti-HA Ab (**b**). Sub-confluent DU145 cells were lysed and subjected to IP with anti-SAG (**c**), anti-PHLPP1 (**d**) or anti-DEPTOR Ab (**e**), along with IgG control, followed by IB with indicated Abs. (**f** & **g**) SAG overexpression shortens protein half-lives of PHLPP1 and DEPTOR: DU145 cells were transfected with HA-PHLPP11or FLAG-DEPTOR, along with the vector control or FLAG-SAG for 48 hrs. Cells were treated with cycloheximide (CHX; 100 μg/ml) to block new protein synthesis for the indicated time periods, followed by IB analysis. (**h** & **i**) SAG knockdown extends the protein half-lives of PHLPP1 and DEPTOR: DU145 cells were infected with Lt-SAG, along with Lt-Con for 72 hrs. Cells were then treated with CHX (100 μg/ml) for indicated time periods and subjected to IB analysis. Densitometry quantification was performed using ImageJ software with β-actin as the loading control and plotted (bottom panels)

Although it is previously demonstrated that both PHLPP1 and DEPTOR are substrates of SCF^βTrCP^ E3[35, 37–39], direct involvement of SAG, which is one of two RING components of SCF [6], has not been previously shown. We, therefore, determined whether SAG forms a complex with either PHLPP1 or DEPTOR. Co-transfection of epitope-tagged SAG with PHLPP1 or DEPTOR, respectively, followed by immunoprecipitation (IP) and western blotting revealed that SAG forms a complex with PHLPP1 (Fig. 3a) and DEPTOR (Fig. 3b) under overexpressed condition. We further determined whether SAG forms a complex with PHLPP1 or DEPTOR under physiological unstressed condition. Indeed, we found that endogenous SAG forms a complex with both PHLPP1 and DEPTOR, along with βTrCP, Cul1 and Cul5 (Fig. 3c). Consistently, in a reciprocal IP experiment, endogenous PHLPP1 or DEPTOR also pulled down endogenous SAG (Fig. 3d and e), indicating that SAG is a component of SCF E3 ligase for targeted degradation of either PHLPP1 or DEPTOR. We next determined whether SAG shortened the protein half-life of PHLPP1 or DEPTOR. As shown in Figure 3f, the protein half-life of ectopically expressed PHLPP1 is greater than 8 hrs (lane 1-4), which was shortened to ~3 hr upon SAG co-transfection (lane 5-8). Likewise, the protein half-life of ectopically expressed DEPTOR also was shortened by SAG cotransfection (Fig. 3g). On the other hand, siRNA knockdown of SAG extended protein half-life of endogenous PHLPP1 (Fig 3h) or DEPTOR (Fig. 3i) in DU145 cells as well as in PC3 cells (Additional file 1: Figure S4A). Furthermore, we found that MLN4924, a small molecule inhibitor of NEDD8 activating enzyme, which indirectly inhibits CRL E3 ubiquitin ligase by blocking cullin neddylation [15, 46], effectively extended the protein half-life of PHLPP1 and DEPTOR in DU145 (Additional file 1: Figure S4B) and PC3 cells (Additional file 1: Figure S4C).

**Fig. 4:**
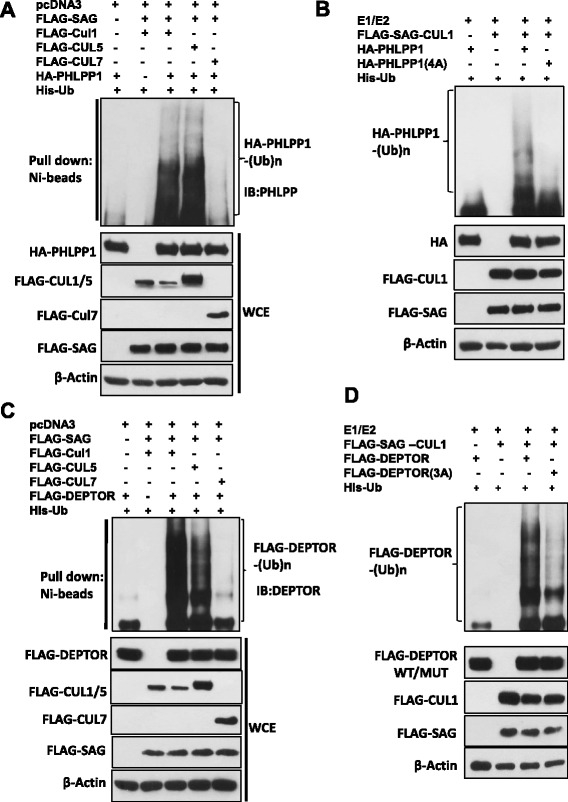
SAG-SCF^β-TrCP^ promotes poly-ubiquitylation of PHLPP1 and DEPTOR: **a** & **c** The *in vivo* ubiquitylation assay: The 293 cells were transfected with indicated plasmids and lysed under denatured condition (6 M guanidinium solution), followed by Ni-bead pull-down. Washed beads were boiled and subjected to IB for PHLPP1 (**a**) or DEPTOR (**c**). **b** & **d** The *in vitro* ubiquitylation assay: SAG-CUL1 E3 was prepared by FLAG-conjugated beads IP using 293 cells transfected with SAG, along with CUL1. PHLPP1 or PHLPP1(4A) was prepared by transfecting HA-PHLPP1 or HA-PHLPP1(4A) into 293 cells, followed by HA-conjugated beads IP and 1× HA peptide elution (**b**) or DEPTOR or DEPTOR(3A) was transfected into 293 cells, followed by FLAG-conjugated beads and 3× FLAG peptide elution (**d**). SCF E3 and substrates (PHLPP1 or DEPTOR) or their mutants were added, respectively, into a reaction mixture containing ATP, ubiquitin, E1 and E2, followed by constant mixing for 60 min. The reaction mixture was boiled with loading buffer and then loaded onto SDS-PAGE gel for IB using PHLPP1 (**b**) or DEPTOR (**d**) Ab

Finally, we determined whether manipulation of SAG levels would alter the polyubiquitylation of PHLPP1 or DEPTOR. Indeed, our *in vivo* ubiquitylation assay showed that overexpression of SAG/CUL1 or SAG/CUL5 promoted poly-ubiquitylation of exogenously expressed PHLPP1 (Fig. 4a) or DEPTOR (Fig. 4c). This effect is rather specificity, since SAG-CUL7 failed to promote such poly-ubiquitylation (Fig. 4a&c). Consistently, in an *in vitro* ubiquitylation assay, addition of purified PHLPP1 or DEPTOR into a reaction mixture containing E1, E2, and SAG-CUL1 E3, co-purified by beads-conjugated immunoprecipitation, induced polyubiquitylation of PHLPP1 (Fig. 4b) or DEPTOR (Fig. 4d), In contrast, SAG-CUL1-induced polyubiquitylation was significantly reduced when the β-TrCP binding motif on PHLPP1 or DEPTOR was mutated (Fig. 4b&d). Taken together, our results demonstrated that PHLPP1 and DEPTOR are indeed substrates of SAG-SCF^β-TrCP^ E3 ubiquitin ligase and SAG inactivation leads to accumulation of PHLPP1 and DEPTOR, as a result of reduced ubiquitylation and degradation.

### Growth suppression by SAG knockdown is partially rescued by knockdown of PHLPP1 or DEPTOR

**Fig. 5:**
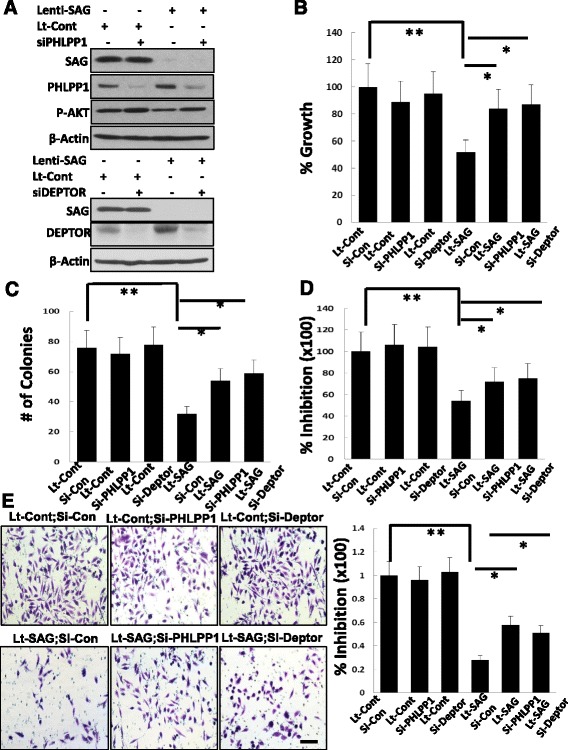
Knockdown of PHLPP1 or DEPTOR partially rescues growth suppression triggered by SAG deletion. DU145 cells were first infected with Lt-SAG to knockdown SAG along with the control (Lt-Con), followed by transfection with PHLPP1 or DEPTOR siRNA oligonucleotides. A portion of cells were harvested for immunoblotting **a**; the other portions for monolayer growth for 4 days, followed by ATP-lite proliferation assay **b**; clonogenic assay for survival **c**; soft agar assay for anchorage-independent growth **d**, or Boyden chamber migration assay **e**. Shown are mean ± SEM from three independent experiments **b**-**e**. * *P* < 0.05; ** *P* < 0.01

We next determined the functional significance of accumulation of PHLPP1 or DEPTOR in mediating growth suppression phenotype induced by SAG knockdown. Indeed, simultaneous knockdown of PHLPP1 or DEPTOR with SAG in DU145 cells (Fig. 5a) partially reversed the growth suppression in monolayer proliferation (Fig. 5b), clonogenic survival (Fig. 5c) anchorage independent growth in soft agar (Fig. 5d), and cell migration (Fig. 5e). Thus, accumulation of PHLPP1 or DEPTOR upon SAG knockdown plays at least in part a causal role in suppression of tumor cell growth and reverse of tumor cell phenotypes.

### *Sag* deletion attenuates the PI3K/AKT/mTOR signaling in mouse prostate tissues

**Fig. 6:**
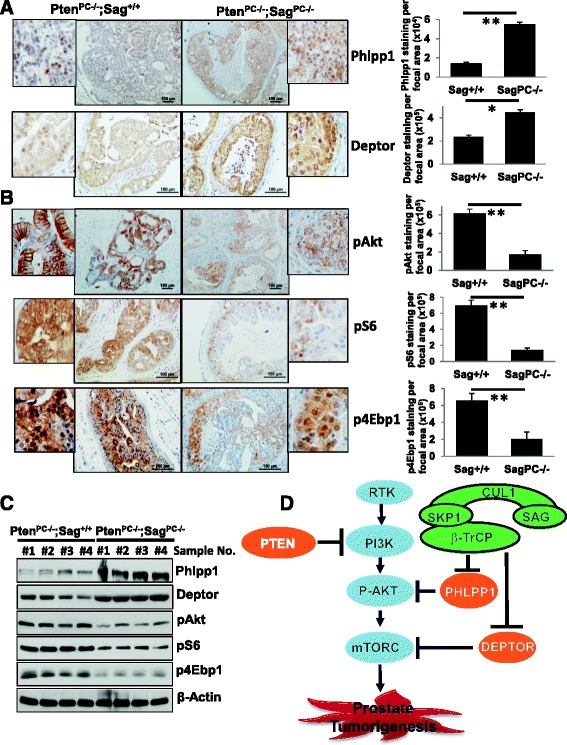
Sag-SCF^βTrCP^ modulates the PI3K/AKT/mTOR axis during mouse prostate tumorigenesis driven by *Pten* loss. **a**-**c** Accumulation of Phlpp1 and Deptor, and reduction of pAkt, pS6 and p4Ebp1: (**a** & **b**) Prostate tissues from mice at age of 6 month with indicated genotypes were stained with indicated Abs. Shown are representative areas of stained tissues (*left panel*), and the staining was analyzed using 3D Histech software and plotted (*right panel*). All values represent means ± SD; n ≥ 5 for each group. *p ≤ 0.05; **p ≤ 0.01. Scale bars: 100 μm. **c** Cell lysates from prostate tissues with indicated genotypes (4 independent samples from two genotypes, respectively) were analyzed by Western blotting with indicated Abs. **d** Working Model: *Sag* deletion blocks prostate tumorigenesis triggered by *Pten* loss. The PI-3 K/AKT/mTOR axis was activated upon *Pten* loss to trigger prostate tumorigenesis, whereas *Sag* deletion caused accumulation of Phlpp1 to inactivate Akt, and of Deptor to inactivate mTOR, leading the suppression of prostate tumorigenesis

Finally, we returned to our mouse prostate cancer model and determined whether *Sag* deletion will indeed block the activation of the PI3K/AKT/mTOR signal triggered by *Pten* loss. By immunohistochemical (IHC) staining and western blotting of prostate tissues from 6 month old mice, we first confirmed that *Sag* deletion indeed caused accumulation of both Phlpp1 and Deptor (Fig. 6a&c). Consequently, we found a significant reduction of pAkt, pS6 and p4Ebp1 in the prostate tissues from the *Pten* ^*PC*-/-^;*Sag* ^*PC*-/-^ mice as compared to that from control *Pten* ^*PC*-/-^;*Sag* ^+/+^ mice (Fig. 6b&c). As a negative control, no change in AR staining was observed between two groups (Additional file 1: Figure S5). Thus, suppression of prostate tumorigenesis triggered by *Pten* loss could be attributable to the accumulation of Phlpp1 and Deptor, as a result of *Sag* deletion.

## Discussion

Abnormal activation of the PTEN/AKT/mTOR pathway is the most frequent event in prostate cancer [47] and it is, therefore, important to identify the cofactors modulating prostate cancer progression in the context of altered PTEN/AKT/mTOR signaling. In this study, we demonstrated that selective depletion of Sag E3 ligase from the mouse prostate epithelial cells is sufficient to delay the progression of prostate tumorigenesis triggered by *Pten* loss. This is achieved through accumulation of PHLPP1 to inactivate AKT and of DEPTOR to inhibit mTOR activity, resulting from reduced ubiquitylation and degradation upon Sag depletion.

Overexpression of a given gene in human cancers does not predict whether its overexpression is causally related to or just the consequence of tumorigenesis. In case of SAG, which is overexpressed progressively in prostate cancer from the early-to-later stages, there has never been mechanistically pursued as to whether SAG is required for prostate cancer initiation and progression, or for the maintenance of prostate cancer cell phenotypes or simply as the consequence of prostate tumorigenesis. Here, we addressed this important issue using a mouse prostate tumorigenesis model, triggered by *Pten* loss, which recapitulates the entire process of human prostate tumorigenesis with sequential formation of lesions such as hyperplasia/LGPIN, HGPIN and eventually adenocarcinoma in a manner dependent on the length of *Pten* inactivation [33]. By the use of a compound mouse model with *Pten* and *Sag* conditional knockout alleles (*Pten* ^*fl*/*fl*^;*Sag* ^*fl*/*fl*^ *)* in which *Pten* and *Sag* deletion occur concomitantly upon Cre expression driven by specific Pb4 promoter in prostate epithelial cells, we showed that *Sag* inactivation remarkably inhibits prostate tumorigenesis, induced by *Pten* loss, as evidenced by delayed disease progression (Fig. 1f & g), and reduced proliferation of cancer cells (Figs. 1i & j and Additional file 1: Figure S1A&B). The suppressive effect of *Sag* deletion is most likely attributable to inactivation of PI3K/AKT/mTOR pathway via accumulation of PHLPP1, a Ser/Thr protein phosphate, that directly dephosphorylates pAkt, and of Deptor, that directly inhibits the mTOR activity (Fig. 6).

To gain mechanistic insight into SAG action, we used the loss-of-function approach in two human prostate cancer cell lines and found that SiRNA-based SAG knockdown caused in general accumulation of PHLPP1 and DEPTOR with consequential inactivation of pAKT and mTORC1 activity (Figs. 2e and Additional file 1: Figure S2E). The effect on PHLPP1 and DEPTOR is rather specific, since no changes were found upon SAG knockdown in the levels of p21 and p27, two other SAG/CRL1 substrates [25, 27]; NRF2, a CRL1/SCF^β-TrCP^ substrate [48, 49], as well as a CRL3 substrate [50]; and two CRL5 substrates, DAB1 [51] and FLNA [52]. Thus, it appears that SAG inactivation selectively accumulates its substrates to suppress prostate tumorigenesis. This notion was further supported by our rescued experiment in which simultaneous knockdown of either PHLPP1 or DEPTOR largely abrogated the growth suppression triggered by SAG knockdown in DU145 prostate cancer cells (Fig. 5).

We further provide direct evidence that PHLPP1 and DEPTOR are indeed the substrates of SAG E3 ubiquitin ligase, as evidenced by (a) formation of SAG-PHLPP1 or SAG-DEPTOR complex under physiological conditions, likely mediated by β-TrCP-Cullin1/5, since SAG directly binds to Cul1/5 (Fig. 3c) [39], and β-TrCP binds to PHLPP1 [35] or DEPTOR [39]; (b) SAG overexpression shortens protein half-lives of PHLPP1 or DEPTOR, whereas SAG knockdown extends them (Fig. 3f-i); (c) SAG promotes polyubiquitination of PHLPP1 or DEPTOR as shown by both *in vivo* or *in vitro* ubiquitylation assays, and (d) pharmaceutical inactivation of SAG E3 by MLN4924 extends the protein half-lives of PHLPP1 and DEPTOR (Additional file 1: Figure S4B&C). Thus, PHLPP1 and DEPTOR are added to a growing list of SAG substrates.

It is worth noting that SAG is the RING ligase core of CRLs, and not directly involved in substrate recruitment. Substrate recognition will solely rely on F-box proteins in CRL1 and SOCS-box containing proteins in CRL5 [14, 15, 53]. In the case of SAG-CUL1, ubiquitylation of PHLPP1 or DEPTOR appears to be mediated by β-TrCP, since mutation at the β-TrCP binding motif abrogated their poly-ubiquitylation (Fig. 4b&d). However, it is not clear which SOCS protein is involved in the case of SAG-CUL5. Involvement of SAG-CUL5 is evidently, given that the fact that SAG binds to endogenous CUL5 (Fig. 3c) and SAG-CUL5 could promote polyubiquitylation of both PHLPP1 and DEPTOR (Fig. 4a&c). Future study is directed to identify and characterize the involving SOCS-containing protein in SAG-CUL5-mediated polyubiquitylation of PHLPP1 and DEPTOR. It is also worth noting that the effect of SAG knockdown (Fig. 5) appears not to be compensated by RBX1 in prostate cancer cells. Our previous gene knockout studies have shown that the effect of SAG and RBX1 is functionally non-redundant during mouse embryogenesis, since total KO of either Sag [17] or *Rbx1* [20] caused embryonic lethality. Our most recent study [54] revealed that SAG and RBX1 form catalytic complex with different E2 enzymes to promote poly-ubiquitylation of respective substrates via K11 or K48-linkage, respectively, which further supports their functional non-redundancy.

## Conclusions

Our study supports the following model. During prostate tumorigenesis, SAG is induced in response to various stresses, such as hypoxia [55], ROS [11], and oncogene activation [25, 43]. Increased SAG facilitates prostate tumorigenesis by promoting ubiquitylation and degradation of Phlpp1 to increase p-Akt, or of Deptor to activate mTor activity, further enhancing the PI3k/Akt/mTor signaling, activated by *Pten* loss. On the other hand, *Sag* deletion antagonizes the PI3k/Akt/mTor signaling by causing accumulation of Phlpp1 and Deptor, leading to inhibition of prostate tumorigenesis (Fig. 6d). Our study, therefore, provides experimental evidence from both *in vivo* animal and *in vitro* cell culture models, suggesting that SAG E3 ligase is an attractive target against prostate cancer derived from *Pten* loss.

Finally, given that *Sag* is pro-oncogenic in the lung [25] and tumor-suppressive in the skin [29] during Kras^G12D^-induced tumorigenesis, and pro-oncogenic in the prostate during tumorigenesis induced by Pten loss (this study), *Sag* appears to be a conditional pro-oncogenic or tumor suppressive co-operating gene in tissue- and context-dependent manner.

## Additional file

Additional file 1: Figure S1.
*Sag* deletion reduced prostate epithelium cell proliferation without affecting apoptosis: (A&B) Prostate tissues with indicated genotypes were labeled by BrdU and representative images were shown. Positive cells were counted from at least 3 randomly selected microscopic fields. * *P*<0.05. Scale bar: 100 μm. (C-F) Prostate lesions were stained for cleaved caspase3 (C) and TUNEL (E) with representative images shown. Cells with positive staining of cleaved caspase3 (D) and TUNEL (F) were counted from at least 3 randomly selective microscopic fields. **Figure S2.** SAG Knockdown suppresses growth, survival and migration of human prostate cancer cells via inactivation of the PI3K/AKT/mTOR axis. PC3 cells were infected with Lenti-SAG or Lenti-GFP for 72 hrs. Cell proliferation was measured by ATP-lite assay (n=3) (A), clonogenic survival (n=3) (B), soft agar assay (n=3) (C), and Boyden chamber migration assay (n=3) (D), as well as western blotting assay using indicated Abs (E). **Figure S3.** SAG Knockdown has no effect on the levels of FLNA, DAB1 and NRF2. Du145 and PC3 cells were infected with Lenti-SAG or Lenti-GFP for 72 hrs. Cells were subjected to IB with indicated antibodies. **Figure S4.** SAG knockdown or MLN4924 treatment extended the protein half-lives of PHLPP1 and DEPTOR. PC3 cells were infected with Lt-SAG, along with Lt-Cont for 72 hrs. Cells were then treated with CHX for indicated time periods and subjected to IB analysis (A). DU145 (B) or PC3 (C) cells were treated with CHX for indicated time periods in the absence or presence of MLN4924. Cells were subjected to IB. Densitometry quantification was performed (right panels for A and bottom panels for B&C). **Figure S5.** *Sag* deletion has no effect on AR expression. Prostate tissues were stained with anti-AR Ab. Shown are representative areas of stained tissues (top panel), and the staining quantification (bottom panel). (PPTX 3263 kb)

## Abbreviation

BrdU: 5-Bromo-2-Deoxyuridine
CHX: Cycloheximide
CRLs: Cullin-RING ubiquitin Ligases
DEPTOR: DEP domain containing mTOR interacting protein
IB: Immunoblotting
IHC: Immuno-histochemistry
IP: Immunoprecipitation
mTOR: mammalian target of rapamycin
PHLPP1: PH domain and Leucine rich repeat Protein Phosphatases
PI3K: Phosphoinositide 3-kinase
PIN: prostate intraepithelial neoplasia
PTEN: Phosphatase and Tensin homolog
ROS: Reactive Oxygen Species
SAG: Sensitive to Apoptosis Gene
SCF: Skp1, Cullins, F-box proteins
siRNA: Small interfering RNA
TUNEL: Terminal deoxynucleotidyl Transferase Biotin-dUTP Nick End Labeling.
β-TrCP: β-transducin repeat-containing protein
