# Supplementary material for: Depletion of SAG/RBX2 E3 ubiquitin ligase suppresses prostate tumorigenesis via inactivation of the PI3K/AKT/mTOR axis
Source: Mol Cancer. 2016 Dec 12;15:81. doi: 10.1186/s12943-016-0567-6 (PMC5153812; doi:10.1186/s12943-016-0567-6)
Supplement: Additional file 1: Figure S1. — Sag deletion reduced prostate epithelium cell proliferation without affecting apoptosis: (A&B) Prostate tissues with indicated genotypes were labeled by BrdU and representative images were shown. Positive cells were counted from at least 3 randomly selected microscopic fields. * P<0.05. Scale bar: 100 μm. (C-F) Prostate lesions were stained for cleaved caspase3 (C) and TUNEL (E) with representative images shown. Cells with positive staining of cleaved caspase3 (D) and TUNEL (F) were counted from at least 3 randomly selective microscopic fields. Figure S2. SAG Knockdown suppresses growth, survival and migration of human prostate cancer cells via inactivation of the PI3K/AKT/mTOR axis. PC3 cells were infected with Lenti-SAG or Lenti-GFP for 72 hrs. Cell proliferation was measured by ATP-lite assay (n=3) (A), clonogenic survival (n=3) (B), soft agar assay (n=3) (C), and Boyden chamber migration assay (n=3) (D), as well as western blotting assay using indicated Abs (E). Figure S3. SAG Knockdown has no effect on the levels of FLNA, DAB1 and NRF2. Du145 and PC3 cells were infected with Lenti-SAG or Lenti-GFP for 72 hrs. Cells were subjected to IB with indicated antibodies. Figure S4. SAG knockdown or MLN4924 treatment extended the protein half-lives of PHLPP1 and DEPTOR. PC3 cells were infected with Lt-SAG, along with Lt-Cont for 72 hrs. Cells were then treated with CHX for indicated time periods and subjected to IB analysis (A). DU145 (B) or PC3 (C) cells were treated with CHX for indicated time periods in the absence or presence of MLN4924. Cells were subjected to IB. Densitometry quantification was performed (right panels for A and bottom panels for B&C). Figure S5. Sag deletion has no effect on AR expression. Prostate tissues were stained with anti-AR Ab. Shown are representative areas of stained tissues (top panel), and the staining quantification (bottom panel). (PPTX 3263 kb) [file 12943_2016_567_MOESM1_ESM.pptx]

## Slide 1
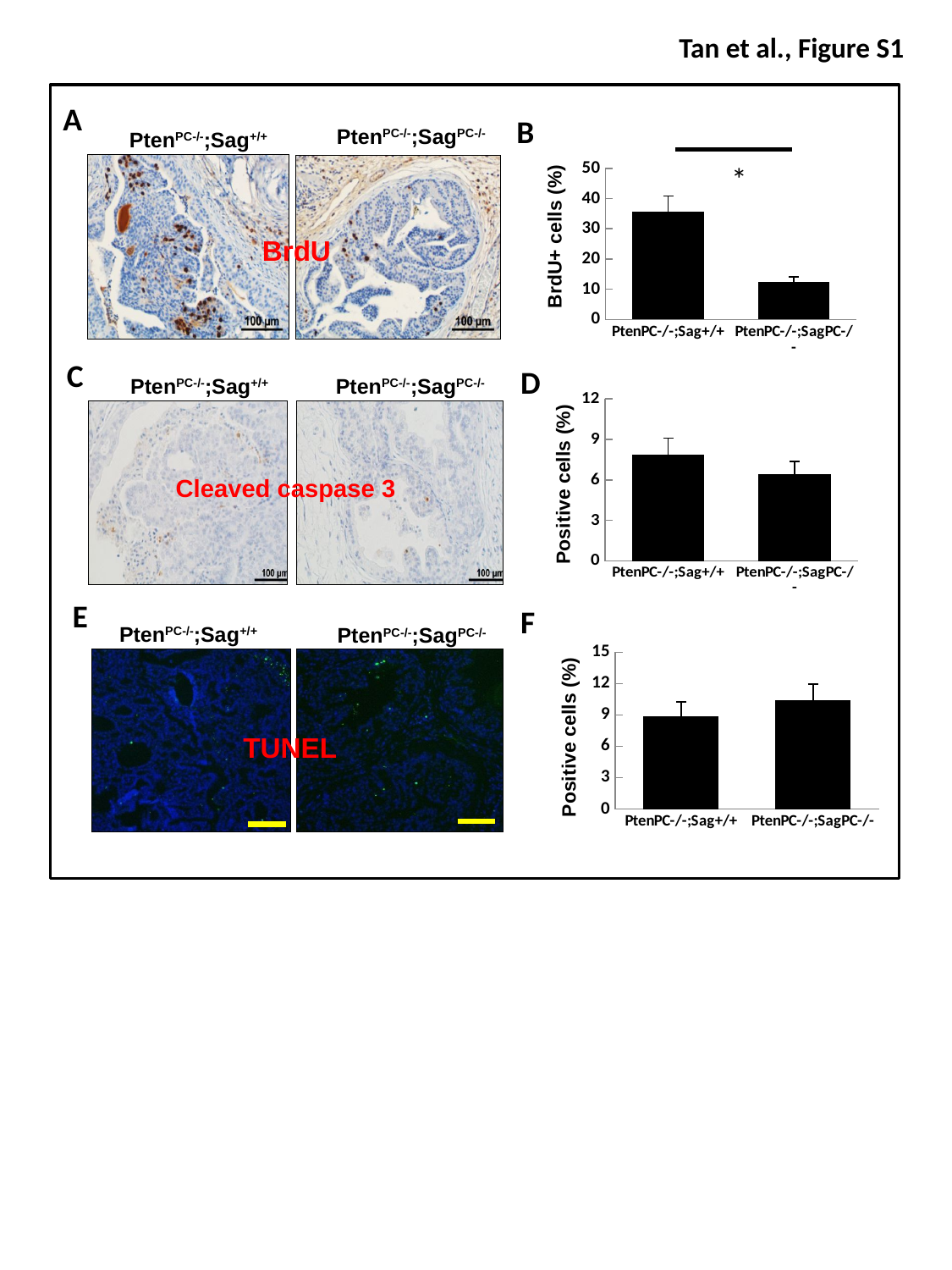

Tan et al., Figure S1
A
B
PtenPC-/-;SagPC-/-
PtenPC-/-;Sag+/+
### Chart
| Category | |
|---|---|
| PtenPC-/-;Sag+/+ | 35.8 |
| PtenPC-/-;SagPC-/- | 12.4 |*
BrdU+ cells (%)
BrdU
C
D
PtenPC-/-;Sag+/+
PtenPC-/-;SagPC-/-
### Chart
| Category | |
|---|---|
| PtenPC-/-;Sag+/+ | 7.9 |
| PtenPC-/-;SagPC-/- | 6.4 |
Positive cells (%)
Cleaved caspase 3
E
F
PtenPC-/-;Sag+/+
PtenPC-/-;SagPC-/-
### Chart
| Category | |
|---|---|
| PtenPC-/-;Sag+/+ | 8.9 |
| PtenPC-/-;SagPC-/- | 10.4 |
Positive cells (%)
TUNEL

## Slide 2
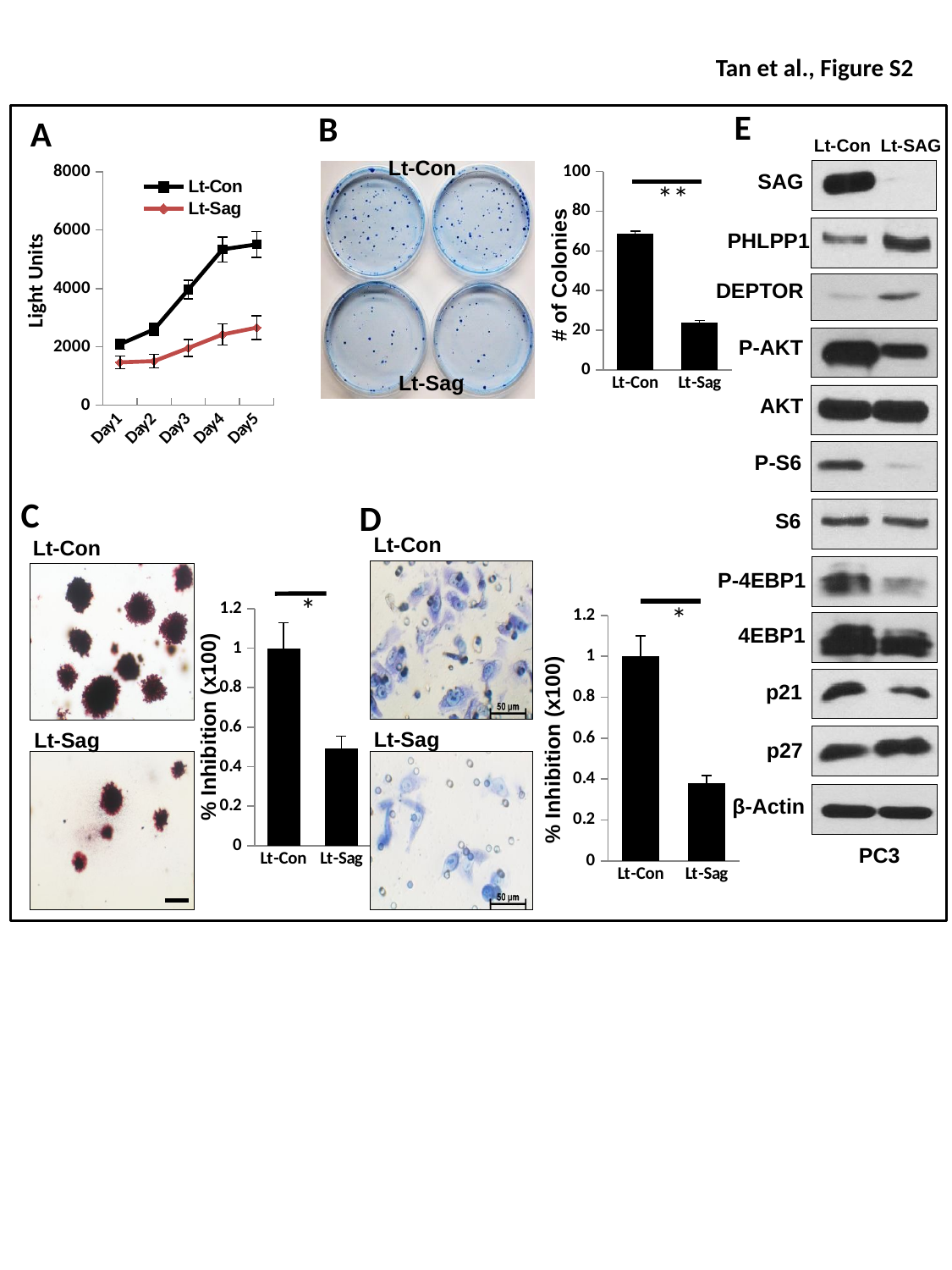

Tan et al., Figure S2
E
B
A
Lt-Con Lt-SAG
Lt-Con
### Chart
| Category | Lt-Con | Lt-Sag |
|---|---|---|
| Day1 | 2091.0 | 1470.0 |
| Day2 | 2597.0 | 1513.0 |
| Day3 | 3958.0 | 1960.0 |
| Day4 | 5339.0 | 2423.0 |
| Day5 | 5508.0 | 2658.0 |
### Chart
| Category | |
|---|---|
| Lt-Con | 68.7 |
| Lt-Sag | 24.0 |**
# of Colonies
SAG
PHLPP1
Light Units
DEPTOR
P-AKT
Lt-Sag
AKT
P-S6
C
D
S6
Lt-Con
Lt-Con
P-4EBP1
*
### Chart
| Category | |
|---|---|
| Lt-Con | 1.0 |
| Lt-Sag | 0.49000000000000005 |% Inhibition (x100)
*
### Chart
| Category | |
|---|---|
| Lt-Con | 1.0 |
| Lt-Sag | 0.38000000000000006 |
4EBP1
p21
Lt-Sag
Lt-Sag
% Inhibition (x100)
p27
β-Actin
PC3

## Slide 3
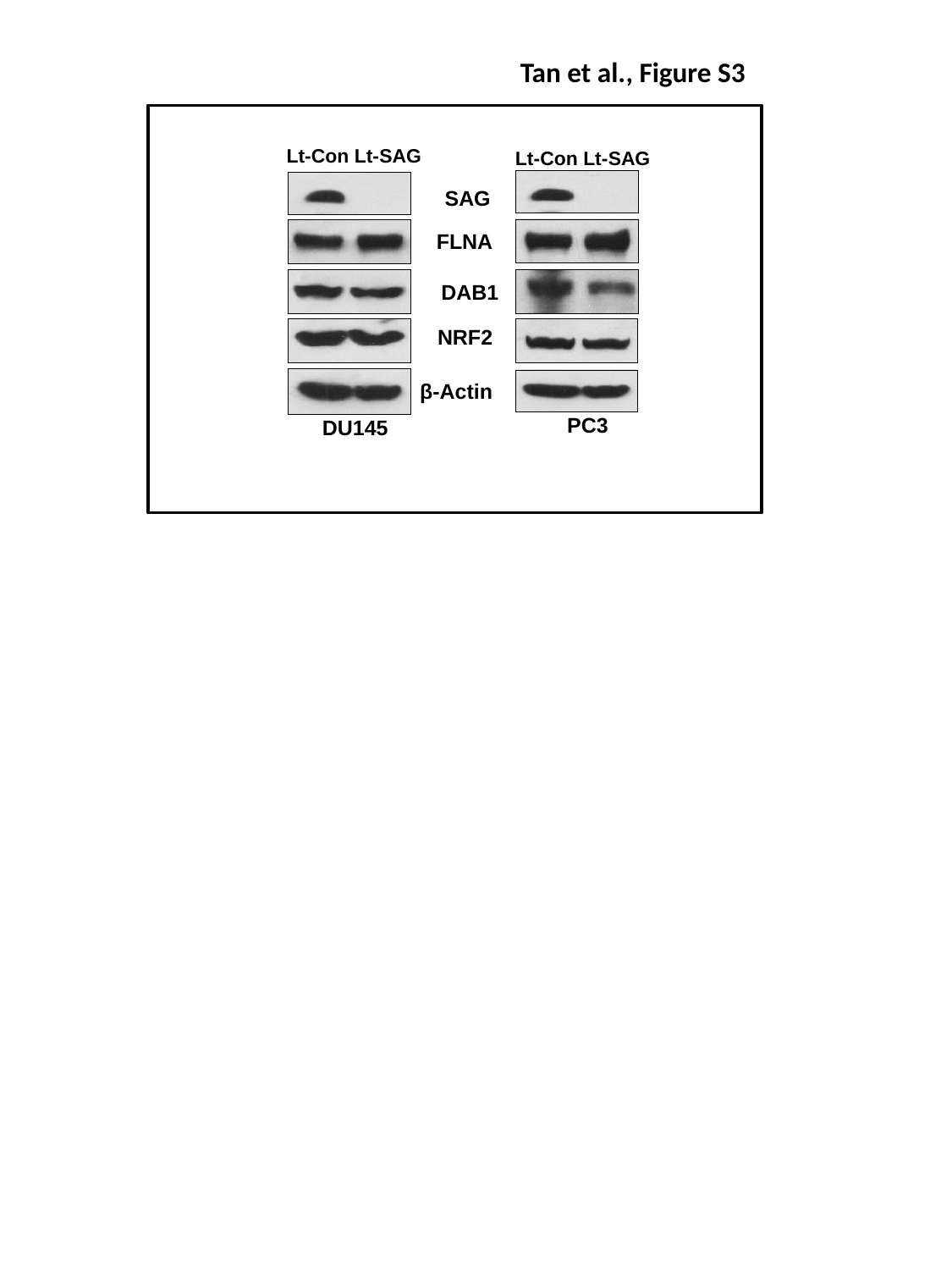

Tan et al., Figure S3
Lt-Con Lt-SAG
Lt-Con Lt-SAG
SAG
FLNA
DAB1
NRF2
β-Actin
PC3
DU145

## Slide 4
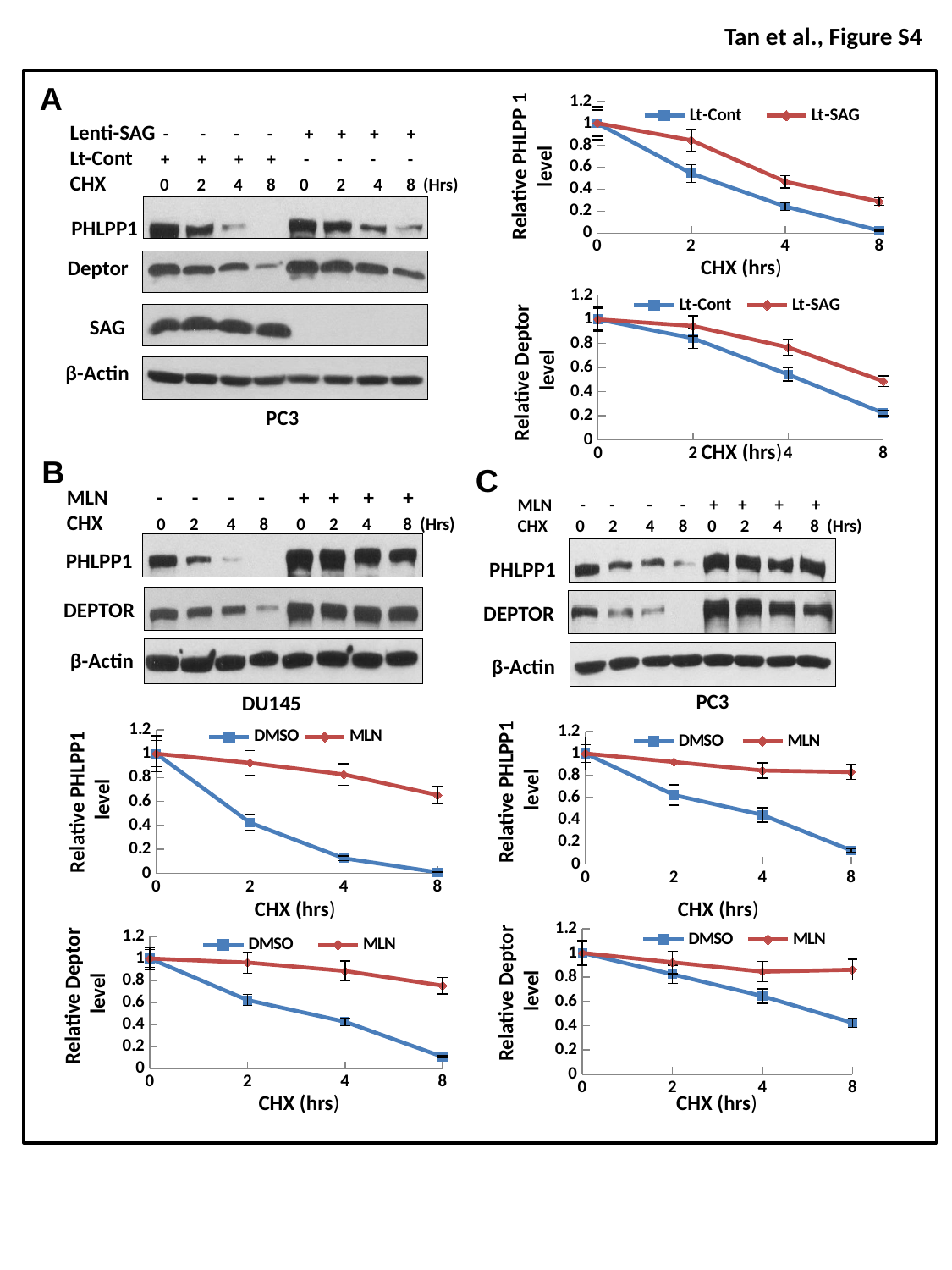

Tan et al., Figure S4
A
### Chart
| Category | Lt-Cont | Lt-SAG |
|---|---|---|
| 0 | 1.0 | 1.0 |
| 2 | 0.543 | 0.8450000000000001 |
| 4 | 0.24300000000000002 | 0.4680000000000001 |
| 8 | 0.023 | 0.28700000000000003 |Lenti-SAG - - - - + + + +
Lt-Cont + + + + - - - -
CHX 0 2 4 8 0 2 4 8 (Hrs)
Relative PHLPP 1
level
PHLPP1
CHX (hrs)
Deptor
### Chart
| Category | Lt-Cont | Lt-SAG |
|---|---|---|
| 0 | 1.0 | 1.0 |
| 2 | 0.843 | 0.945 |
| 4 | 0.543 | 0.768 |
| 8 | 0.223 | 0.487 |
SAG
Relative Deptor
 level
β-Actin
PC3
CHX (hrs)
B
C
MLN - - - - + + + +
CHX 0 2 4 8 0 2 4 8 (Hrs)
MLN - - - - + + + +
CHX 0 2 4 8 0 2 4 8 (Hrs)
PHLPP1
PHLPP1
DEPTOR
DEPTOR
β-Actin
β-Actin
PC3
DU145
### Chart
| Category | DMSO | MLN |
|---|---|---|
| 0 | 1.0 | 1.0 |
| 2 | 0.6240000000000001 | 0.923 |
| 4 | 0.444 | 0.8460000000000001 |
| 8 | 0.12400000000000001 | 0.8320000000000001 |
### Chart
| Category | DMSO | MLN |
|---|---|---|
| 0 | 1.0 | 1.0 |
| 2 | 0.4230000000000001 | 0.923 |
| 4 | 0.126 | 0.8270000000000001 |
| 8 | 0.008000000000000002 | 0.6540000000000001 |Relative PHLPP1
 level
Relative PHLPP1
 level
CHX (hrs)
CHX (hrs)
### Chart
| Category | DMSO | MLN |
|---|---|---|
| 0 | 1.0 | 1.0 |
| 2 | 0.824 | 0.923 |
| 4 | 0.644 | 0.846 |
| 8 | 0.424 | 0.862 |
### Chart
| Category | DMSO | MLN |
|---|---|---|
| 0 | 1.0 | 1.0 |
| 2 | 0.623 | 0.963 |
| 4 | 0.426 | 0.887 |
| 8 | 0.108 | 0.754 |Relative Deptor
 level
Relative Deptor
level
CHX (hrs)
CHX (hrs)

## Slide 5
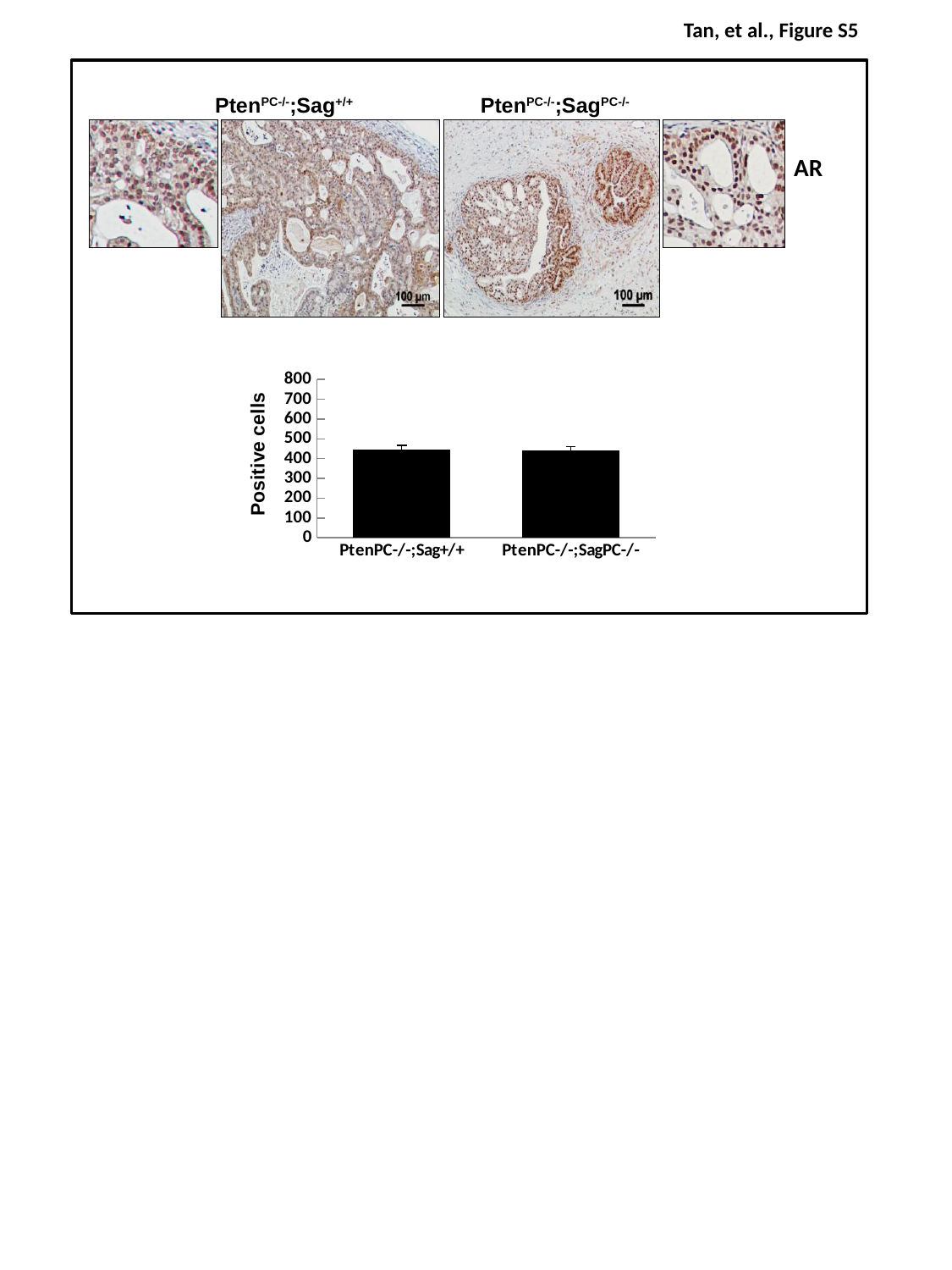

Tan, et al., Figure S5
PtenPC-/-;Sag+/+
PtenPC-/-;SagPC-/-
AR
### Chart
| Category | |
|---|---|
| PtenPC-/-;Sag+/+ | 445.0 |
| PtenPC-/-;SagPC-/- | 441.0 |Positive cells
